# District Court—Judge Hare.—The Use of Chloroform

**Published:** 1863-02

**Authors:** 


District Court—Judge Hare.—The Use of Chloroform.—John P. Bogle *vs.* Henry G. Winslow. Before reported. The features of this case are so interesting, and the subject they concern of such importance to the community, that we have been led to prepare a rather extended report of the case. Our notice yesterday was a brief one, because we were not then in possession of all the facts of the case. We may now state it as follows:

On the 21st of April last, the plaintiff, a driver on the Tenth and Eleventh streets railway, who was suffering with the tooth-ache, was induced by the recommendation of a lady friend to call upon the defendant for the purpose of having some teeth and the roots of decayed others extracted, while under the influence of chloroform. Dr. Winslow was recognized by the profession as one eminently skilful in the use of this anesthetic agent, and patients were frequently taken to him by other physicians.

On the day named, Sunday, the plaintiff accordingly called on him, and the chloroform was administered and the roots extracted. Before this had been successfully accomplished, however, it was found to be necessary to administer the chloroform in large quantities, and for three quarters of an hour, and in the intervals between the drawing of the different teeth and roots, as signs of returning consciousness appeared.

The operation completed, Mr. Bogle left, in company with his lady friend, but the influence of the volatile fluid was still apparent. He staggered like a drunken man, and was obliged to lean on his companion for assistance. He grew worse after that; his tongue thickened so that his articulation became indistinct, and finally, on the fourth day, he was struck with paralysis of the left side.

Dr. Winslow was called in, and treated him for this sickness for four weeks, at the end of which time, no perceptible relief having been afforded, another physician, Dr. Longshore, was employed, under whose treatment he remained until the 24th of July, when he was able partially to resume his employment—acting as substitute conductor. For the loss sustained by him by reason of his sickness and continued inability to attend to his duties this suit was instituted.

Dr. Longshore, who was examined, testified that, after hearing the testimony in the case, he inferred that the chloroform was the cause of the paralysis; never knew chloroform to be given without producing paralysis; that is its purpose; it is not permanent, however; there are cases reported in the books of paralysis of the tongue, resulting from the use of chloroform; never heard of a case of paralysis of the side; chloroform might produce paralysis of the side by reason of its effect upon the brain; cerebral hæmorrhage produces paralysis; never heard of a case of cerebral hæmorrhage produced by chloroform; the kick of a horse will produce it; do not think it resulted from such a cause in this case; there is no standard for a dose of chloroform; the operator must be governed by the action of the patient; if the effect was not produced in three quarters of an hour I would stop the use of the chloroform, as I would be afraid of the consequence; there might have been an injury to the brain, brought into action by the use of chloroform, but if it resulted from injuries received two months before, there would be complaints on the part of the patient.

Dr. Harbeson, who was also called for the plaintiff, testified that in his experience he knew of a case where paralysis was caused by the use of chloroform. A tumor had been removed from the left breast of a patient, while under the influence of chloroform, and paralysis ensued. He also testified that it was considered a dangerous agent, and was, for that reason, not used at the Pennsylvania Hospital. He had known of death resulting from its use.

The defense set up was, that Dr. Winslow was a graduate of twenty years' practice, eminently skilled in the use of chloroform, and that no matter how large the quantity used, or the length of its application, no such effect as paralysis could result. Besides it was on evidence that in the preceding January the plaintiff had been kicked in the breast by one of his car horses, hurled over the dasher into the street, and against the curb, his head violently striking a lamp-post, and it was contended that this was more likely to have been the cause of the paralysis.

Eminent physicians, among them Drs. Gross and Goddard, were called in support of these allegations. Dr. Gross' testimony embodies the whole, and we present it in substance as follows:

Dr. S. D. Gross, Professor of Surgery at the Jefferson College, testified that chloroform is regarded by the profession in general as a proper agent to relieve pain; it is one of the approved remedies of the profession; in the present case he considered the length of time resulted from the want of the proper number of assistants by Dr. Winslow; don't think there is any case on record, except two referred to by Dr. Longshore, that chloroform caused paralysis; these two are cases reported by Dr. Haphold of South Carolina, and these cases are not authentic; I have given chloroform since 1842, and under almost all circumstances, to a child of six weeks of age, and to a person of seventy-five years of age; I have given it to all classes and never witnessed any ill effects from it; I do not think that the paralysis in this case was the result of the use of chloroform; in my judgment it had nothing to do with it.

Dr. Gross then explained the effect of a concussion of the brain in producing paralysis; several months might elapse between the injury and the paralysis; I think it not unlikely that the patient would complain of headache, etc., though it does not follow that he would actually complain; if the man had not been kicked by a horse I would not attribute the paralysis to chloroform from what I know of its effects. Dr. Snow, of London, in a late work issued by him, states that he had administered chloroform for fourteen years, and he never knew any permanent ill effects to be produced by it; I have given several ounces to patients; in Louisville I gave a man eight ounces, and kept him under its influence for three hours; I have every confidence in Dr. Winslow's skill.

Cross-examined.—Chloroform, like many other agents which a physician is obliged to use, is dangerous; so is laudanum, etc.

Question. If improperly used would it not produce paralysis?

Answer. No, sir.

Question. Would it be improper to continue the use of chloroform after a patient has resisted its influence for nearly three-quarters of an hour?

Answer. No, sir; I should continue for five hours until I accomplished my object; I have taken chloroform myself; a patient who resists only proves that he has not taken enough.

Judge Hare left the question of negligence or unskilfulness to the jury, and they found a verdict for the defendant. The case was very ably managed on both sides, and enlisted the greatest attention. J. P. O'Neill, Esq., appeared for the plaintiff, and D. Webster, Esq., for defendant.—*Philadelphia Press.*

